# TRAF3 mediates neuronal apoptosis in early brain injury following subarachnoid hemorrhage via targeting TAK1-dependent MAPKs and NF-κB pathways

**DOI:** 10.1038/s41419-020-03278-z

**Published:** 2021-01-07

**Authors:** Yan Zhou, Tao Tao, Guangjie Liu, Xuan Gao, Yongyue Gao, Zong Zhuang, Yue Lu, Han Wang, Wei Li, Lingyun Wu, Dingding Zhang, Chunhua Hang

**Affiliations:** 1 Department of Neurosurgery, Nanjing Drum Tower Hospital, The Affiliated Hospital of Nanjing University Medical School, Zhongshan Road 321, 210008 Nanjing, Jiangsu People’s Republic of China; 2 Department of Neurosurgery, Nanjing Drum Tower Hospital, Clinical College of Nanjing Medical University, Zhongshan Road 321, 210008 Nanjing, Jiangsu People’s Republic of China; 3 Department of Neurosurgery, Nanjing Drum Tower Hospital, Clinical Medical College of Southern Medical University (Guangzhou), 210008 Nanjing, Jiangsu People’s Republic of China

**Keywords:** Apoptosis, Neuro-vascular interactions

## Abstract

Neuronal apoptosis has an important role in early brain injury (EBI) following subarachnoid hemorrhage (SAH). TRAF3 was reported as a promising therapeutic target for stroke management, which covered several neuronal apoptosis signaling cascades. Hence, the present study is aimed to determine whether downregulation of TRAF3 could be neuroprotective in SAH-induced EBI. An in vivo SAH model in mice was established by endovascular perforation. Meanwhile, primary cultured cortical neurons of mice treated with oxygen hemoglobin were applied to mimic SAH in vitro. Our results demonstrated that TRAF3 protein expression increased and expressed in neurons both in vivo and in vitro SAH models. TRAF3 siRNA reversed neuronal loss and improved neurological deficits in SAH mice, and reduced cell death in SAH primary neurons. Mechanistically, we found that TRAF3 directly binds to TAK1 and potentiates phosphorylation and activation of TAK1, which further enhances the activation of NF-κB and MAPKs pathways to induce neuronal apoptosis. Importantly, TRAF3 expression was elevated following SAH in human brain tissue and was mainly expressed in neurons. Taken together, our study demonstrates that TRAF3 is an upstream regulator of MAPKs and NF-κB pathways in SAH-induced EBI via its interaction with and activation of TAK1. Furthermore, the TRAF3 may serve as a novel therapeutic target in SAH-induced EBI.

## Introduction

Subarachnoid hemorrhage (SAH) is a major cause of morbidity and mortality in neurosurgery practice^1,2^. Early brain injury (EBI) is regarded as the main factor for poor prognosis after SAH^3,4^. Neuronal apoptosis has been demonstrated to have a vital role in EBI^5,6^. Although inhibition of a single pathway could reduce neuronal apoptosis and improve the prognosis in experimental SAH, few of these research results have been translated into clinical practice^7,8^. Accordingly, targeting multiple neuronal apoptosis pathways simultaneously is currently considered to be a novel treatment strategy for EBI.

Mitogen-activated protein kinases (MAPKs) involving c-Jun-N-terminal kinases (JNK), P38, and nuclear factor-kappa B (NF-κB) signaling pathways have been demonstrated to have an important role in the development of EBI after SAH^9–11^. Specifically, they have been shown to aggravate brain damage by initiating apoptotic pathways^11^. Therefore, therapies that can regulate both MAPKs and NF-κB pathways are superior to single-target agents.

The tumor necrosis factor receptor-associated factor (TRAF) family has been shown to mediate signal transduction of the IL-1 superfamily, TLRs, and TNF receptors^12^. TRAF3, one of the seven members of the TRAF family, deemed as a general immune-related signal transduction regulator, has a variety of biological functions. In some neurological diseases, TRAF3 serves its multiple purposes via interacting with diverse regulatory proteins, kinases, and receptors^13^. Previous research has revealed that neuronal expression of TRAF3 could be induced by ischemic injury, and TRAF3 activated the NF-κB and JNK pathways through regulation of TAK1^14^. Our previous research has demonstrated that TAK1 was a key upstream mediator of neuronal apoptosis in EBI following SAH^15^. Therefore, we hypothesize that TRAF3 might also have an important role in regulating the complex pathological mechanisms of SAH-induced EBI.

## Materials and methods

### Animals

Healthy adult male C57BL/6J mice (22–25 g) and pregnant C57BL/6J mice at 13 days of gestation were purchased from the Nanjing Biomedical Research Institute of Nanjing University. All the experiments were carried out under the guidance of the Guide for the Care and Use of Laboratory Animals which was published by NIH and was approved by the Committee for Animal Experimentation at the Drum Tower Hospital.

### SAH model

The endovascular perforation SAH model was used as previously described^16^. A sharpened prolene filament (Ethicon, Inc., Taipei, Taiwan) was inserted into the intracranial ICA via the cervical ICA and then advanced 3 mm further to puncture the bifurcation-level of ICA while the mice were under isoflurane anesthesia (2% in oxygen gas, 400 ml/min). The animals in the sham group underwent similar operations, with the omission of ICA puncture.

### Exclusion criteria

The neurological score was blindly evaluated by an independent observer using a Modified Garcia scale score 24 h post-SAH^17^. Mice with a Modified Garcia scale score ≤6 or ≥15 were excluded^17^.

### Experimental protocol

**Fig. 1:**
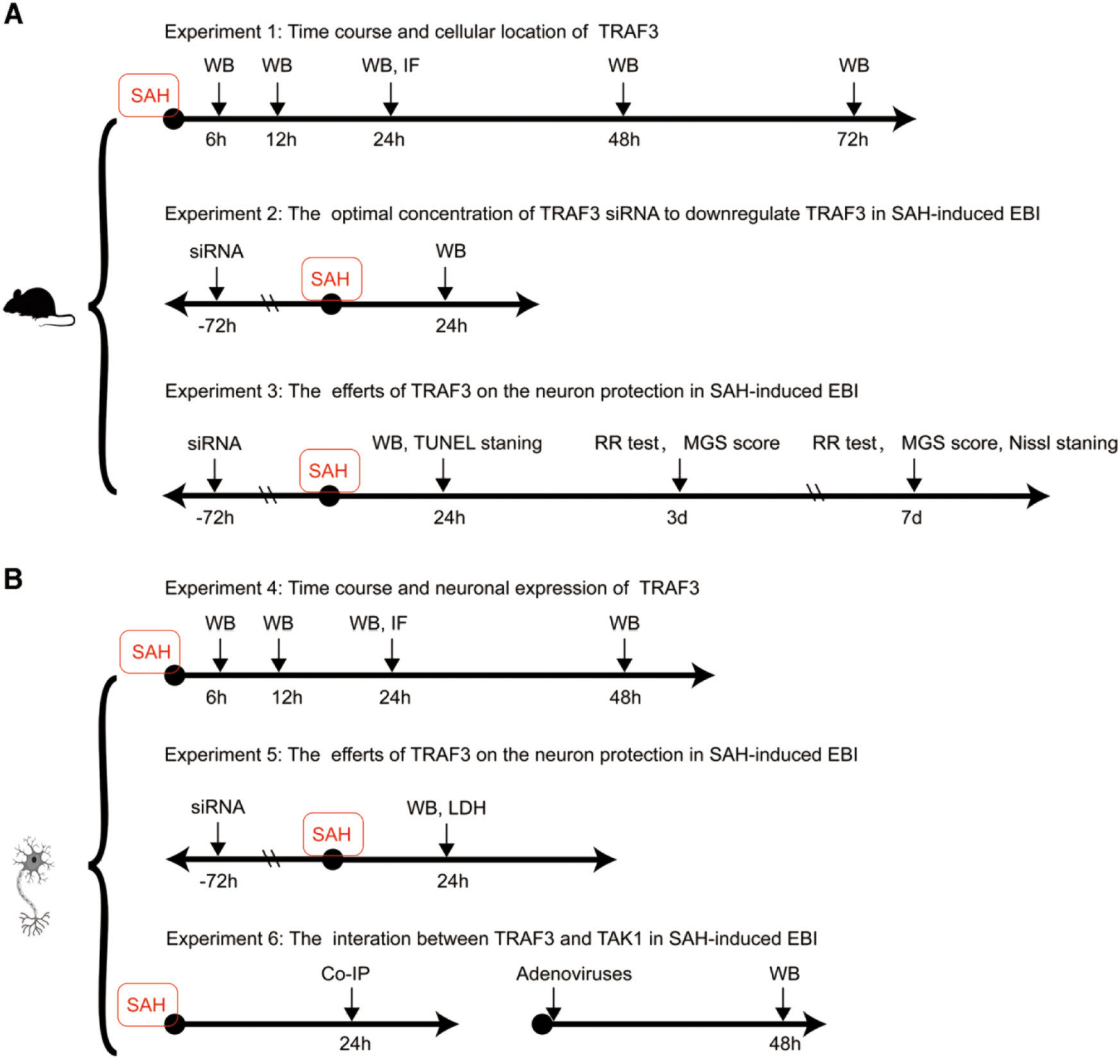
The experimental designs to examine the antiapoptotic role of TRAF3 in SAH-induced EBI. The experimental designs in vivo (**A**) and in vitro (**B**). WB western blotting, IF immunofluorescence staining, MGS Modified Garcia Scale, siRNA control siRNA, and TRAF3 siRNA; Co-IP co-immunoprecipitation; adenovirus AdTRAF3, AdTRAF3-M, and AdGFP.

All mice were randomly assigned to the following experiments as described (Fig. 1).

#### Experimental design 1

Animals were randomly assigned to six groups: sham (*n* = 12), 6 h post-SAH (*n* = 6), 12 h post-SAH (*n* = 6), 24 h post-SAH (*n* = 12), 48 h post-SAH (*n* = 6), and 72 h post-SAH (*n* = 6). At the scheduled time points, tissue was harvested for evaluation of TRAF3 expression (*n* = 6 each). Cellular distribution of TRAF3 was determined by double-staining immunofluorescence in 24 h post-SAH and sham groups (*n* = 6 each).

#### Experimental design 2

Mice were randomly allocated into six groups: (1) sham group (*n* = 6), in which mice underwent surgery without ICA puncture; (2) SAH group (*n* = 6), in which mice underwent ICA puncture; (3) SAH + control siRNA group (*n* = 6), in which mice underwent ICA puncture and intraventricular injection of control siRNA; (4) SAH + 125 pmol of TRAF3 siRNA group (*n* = 6), in which mice underwent ICA puncture and intraventricular injection of 125 pmol of TRAF3 siRNA; (5) SAH + 250 pmol of TRAF3 siRNA group (*n* = 6), in which mice underwent ICA puncture and intraventricular injection of 250 pmol of TRAF3 siRNA; (6) SAH + 500 pmol of TRAF3 siRNA group (*n* = 6), in which mice underwent ICA puncture and intraventricular injection of 500 pmol of TRAF3 siRNA. Brain tissue was harvested for evaluation of TRAF3 expression 24 h post-SAH via western blotting.

#### Experimental design 3

Mice were randomly allocated into four groups: (1) sham group (*n* = 22), in which mice underwent surgery without ICA puncture; (2) SAH group (*n* = 22), in which mice underwent ICA puncture; (3) SAH + control siRNA group (*n* = 22), in which mice underwent ICA puncture and intraventricular injection of control siRNA; (4) SAH + TRAF3 siRNA (*n* = 22), in which mice underwent ICA puncture and intraventricular injection of 250 pmol of TRAF3 siRNA. Out of each group, 12 mice were sacrificed to carry out western blotting and TUNEL staining to determine the effect of TRAF3 in SAH-induced EBI 24 h post-SAH. The remaining mice were assessed using the rotarod test and Modified Garcia scale score on days 3 and 7 following SAH and then sacrificed prior to Nissl staining.

### Primary neuronal cell culture

Primary neuronal cell cultures were prepared from the cortex of fetal C57BL/6 J mice at 13 days of gestation as a modification of a previously described method^18^. Cells were seeded in poly-d-lysine-coated plates (Sigma, Darmstadt, Germany) with neurobasal medium containing 0.5 mM GlutaMax-I (Gibco Company, USA) and 2% B27 supplement (Gibco Company, USA). The medium was replaced 4 h after seeding. Subsequently, half of the medium was replaced every 3 days following seeding. For the in vitro SAH model, cells were incubated with hemoglobin (Millipore Sigma, Darmstadt, Germany) for 24 h at a concentration of 25 μM. Primary neuronal cell cultures were randomly assigned to different groups.

### Patient recruitment and brain tissue collection

All patients in the study were admitted to the Department of Neurosurgery of the Nanjing Drum Tower Hospital between January 2017 and July 2019. Clinical variables are shown in Tables 1 and 2. A total of 4 patients with deep brain tumors that needed surgical resection were selected as control. The samples were obtained from the pathway during surgical removal of the deep tumors. In addition, 4 SAH patients with encephalocele who required surgery were recruited. Written informed consent was obtained from all patients or their family members. The study was conducted in accordance with the Declaration of Helsinki and was approved by the Ethics Committee of Drum Tower Hospital. The team blinded to the clinical parameters of the patients performed the immunofluorescence analysis.

**Table 1:** Descriptions of SAH patients.

Patient	Age (years)	Sex	GCS	Time of surgery from SAH (h)
S1	56	Male	5	12
S2	55	Male	5	15
S3	62	Female	6	7
S4	48	Female	7	20

*GCS* Glascow coma scale score.

**Table 2:** Descriptions of control patients.

Control patient	Age (years)	Sex	Diagnosis	GCS
P1	71	Male	Glioblastoma	15
P2	67	Female	Glioblastoma	15
P3	70	Male	Glioblastoma	15
P4	49	Female	Glioblastoma	15

### SiRNA transfection

Transfection of siRNA with i-Fect siRNA Delivery Kits (NI35150, Neuromics) was carried out to achieve TRAF3 downregulation. The TRAF3 siRNA (SC-36712, Santa Cruz Biotech) and control siRNA (SC-37007, Santa Cruz Biotech) were mixed with i-Fect siRNA Delivery Kits according to the manufacturer’s instructions. In the in vivo experiments, the siRNA was injected into the right lateral ventricles using a Hamilton microsyringe. In the in vitro experiments, the siRNA was mixed with neurobasal medium. The siRNA was transfected 72 h before the SAH inducing procedure^19^.

### Adenoviral vector construction and neuronal infection

We constructed adenoviruses carrying sequences encoding mouse TRAF3 (AdTRAF3), mutated TRAF3 with a mutant 267–376 aa domain (AdTRAF3-M)^14^. AdGFP was used as control. Primary neurons were infected with the adenovirus at a multiplicity of infection (MOI) of 100 for 48 h prior to SAH.

### Western blotting

Brain tissue and primary neural cells were harvested and then homogenized and lysed with RIPA buffer (ThermoFisher Scientific) and phosphatase inhibitor (ThermoFisher Scientific) as previously described. Protein extracts were resolved by 7.5%~12.5% SDA-PAGE (EpiZyme Scientific) and transferred to the Immobilon-FL membranes (Millipore Sigma, Darmstadt, Germany). Then, membranes were incubated with the following primary antibodies at 4°C overnight: anti-TRAF3 (1:500, G-6, Santa Cruz Biotech), anti-P38 (1:1000, 8690, Cell Signaling Technology), anti-P44/42 (1:1000, 4695, Cell Signaling Technology), anti-JNK (1:1000, 9252, Cell Signaling Technology), anti-p-P38 (1:1000, 4511, Cell Signaling Technology), anti-p-P44/42 (1:1000, 4370, Cell Signaling Technology), anti-p-JNK (1:1000, 4668, Cell Signaling Technology), anti-P65 (1:1000, 8242, Cell Signaling Technology), anti-p-P65 (1:1000, 3033, Cell Signaling Technology), anti-IκBα (1:1000, 4812, Cell Signaling Technology), anti-Bax (1:1000, 5023, Cell Signaling Technology), anti-Bcl-xL (1:1000, 2764, Cell Signaling Technology) anti-caspase-3 (1:1000, 9662, Cell Signaling Technology), anti-cleaved caspase-3 (1:1000, 9661, Cell Signaling Technology), anti-TAK1 (1:500, SC-7916, Santa Cruz Biotech), anti-p-TAK1 (1:1000, AF3019, Affinity Biosciences), and anti-GAPDH (1:1000, 5147, Cell Signaling Technology). Then, membranes were incubated with corresponding secondary antibodies (Bioworld). Membranes were visualized using an enhanced chemiluminescence kit (Pekin Elmer, Waltham, MA, USA). The relative band intensities were quantified using ImageJ (National Institutes of Health, Bethesda, MD, USA) software and normalized against GAPDH^20,21^.

### Immunofluorescence

Mice were perfused with PBS and 4% paraformaldehyde after anesthetization. Human brain tissue was postfixed in paraformaldehyde immediately after collection for immunofluorescence staining. Sections of 10 μm were collected from brains after being postfixed in paraformaldehyde for 24 h. Primary neurons seeded in 24-well plates were postfixed in paraformaldehyde for 30 min for immunofluorescence analysis. Cells were stained for TRAF3 (1:100, AB36988, Abcam), NeuN (1:200, ABN78, Millipore), Iba1 (1:200, AB5076, Abcam), GFAP (1:200, AB7260, Abcam) overnight at 4 °C. Primary antibodies were visualized using corresponding secondary antibodies (1:200, Millipore). Immunofluorescence images were captured using the microscope (ZEISS, HB050, Germany)^22^.

### Modified Garcia scale

The Modified Garcia scale contains 6 tests covering spontaneous activity and movement of the four limbs, forepaw outstretching, climbing, body proprioception, and response to whisker stimulation. The 18-point scoring system was applied to assess the functional defects at 1, 3, and 7 days post-SAH by an observer who was blind to groups^23^.

### Rotarod test

The rotarod test was conducted to measure the motor coordination of mice 3 and 7 days post-SAH by an observer who was blind to groups as previously described^24^. Animals were placed on a non-skid rotating rod measuring 3 cm in diameter. The latency (RR time) to fall was measured at 30 rpm for up to 5 min after 6 training sessions given during 3 days^24^.

### TUNEL staining

For terminal deoxynucleotidyl transferase-mediated dUTP nick-end labeling (TUNEL) staining, a TUNEL detection kit (EpiZyme Scientific) was used according to the manufacturer’s instructions. After incubation with a primary antibody against NeuN (1:200, ABN78, Millipore) at 4 °C overnight, the coverslips were incubated with the TUNEL reaction mixture for 45 min and then counterstained using DAPI (Sigma). The positive cells were identified, counted, and analyzed with a fluorescence microscope by two investigators blinded to the grouping.

### Nissl staining

Nissl staining was performed as described in our previous study^25^. Brain sections were stained with cresyl violet and then hydrated in 1% toluidine blue for 10 min. After washing with double-distilled water and mounting with Permount Mounting Medium, the mean number of intact and normal neurons in the views was counted. Six sections from each animal and 6 random high-power views (at ×400 magnification) were chosen for quantification.

### Lactate dehydrogenase (LDH) assay

LDH release detected with the LDH Cytotoxicity Assay Kit (88953, ThermoFisher Scientific) was used to quantify SAH-induced neuronal injury at 24 h post-SAH quantitatively according to the manufacturer’s instructions. Neurons seeded in 96-well plates were incubated with kit reagents. Then, the absorbance at 490 and 680 nm were measured, and LDH units per sample were calculated and expressed as a ratio of LDH released/LDH total (LDH released + LDH extracted post lysis).

### Immunoprecipitation

SAH neurons were harvested and lysed with RIPA buffer (ThermoFisher Scientific). The extracts were precleared and then incubated with 1 μg of TRAF3 antibody (G-6 Santa Cruz Biotech), TAK1 antibody (SC-7916, Santa Cruz Biotech), normal mouse IgG (sc-2025, Santa Cruz Biotech) and 10 μl of protein A/G-agarose (Millipore Sigma, Darmstadt, Germany) beads on a rocking platform at 4 °C overnight. The immunocomplex was collected, washed, and blotted using the TAK1 antibody (1:500, SC-7916, Santa Cruz Biotech) and TRAF3 antibody (1:500, G-6 Santa Cruz Biotech)^14^.

### Statistical analysis

Statistical analysis was performed using Prism (GraphPad Software, La Jolla, CA, USA). All data were first tested for normality via Kolmogorov–Smirnov test. One-way ANOVA analysis was used to analyze differences among multiple groups, followed by Tukey’s post hoc test. The TRAF3 expression of immunofluorescence image was statistically analyzed using the Student’s *t*-test. Values of *p* < 0.05 were considered as statistically significant.

## Results

### Mortality and exclusion

The overall mortality of SAH mice was 17.5% (28/160). No mice died in the sham group. According to the modified Garcia scores on postoperative day 1, a total of 9 mice were excluded from this study (Table 3).

**Table 3:** Mortality and exclusion.

Groups	Mortality rate	Excluded
*Experiment 1*		
Sham	0 (0/12)	0
SAH (6 h, 12 h, 24 h, 48 h, 72 h)	20% (9/45)	3
*Experiment 2*		
Sham	0 (0/6)	0
SAH	25% (2/8)	1
SAH + control siRNA	0 (0/6)	0
SAH + 125 pmol TRAF3 siRNA	14.29% (1/7)	1
SAH + 250 pmol TRAF3 siRNA	14.29% (1/7)	1
SAH + 500 pmol TRAF3 siRNA	25% (2/8)	0
*Experiment 3*		
Sham	0 (0/22)	0
SAH	18.52% (5/27)	1
SAH + control siRNA	13.79% (4/26)	1
SAH + TRAF3 siRNA	13.79% (4/26)	1
Total		
Sham	0 (0/40)	0
SAH	17.5% (28/160)	9

### Expression of TRAF3 in the cortex after SAH

**Fig. 2:**
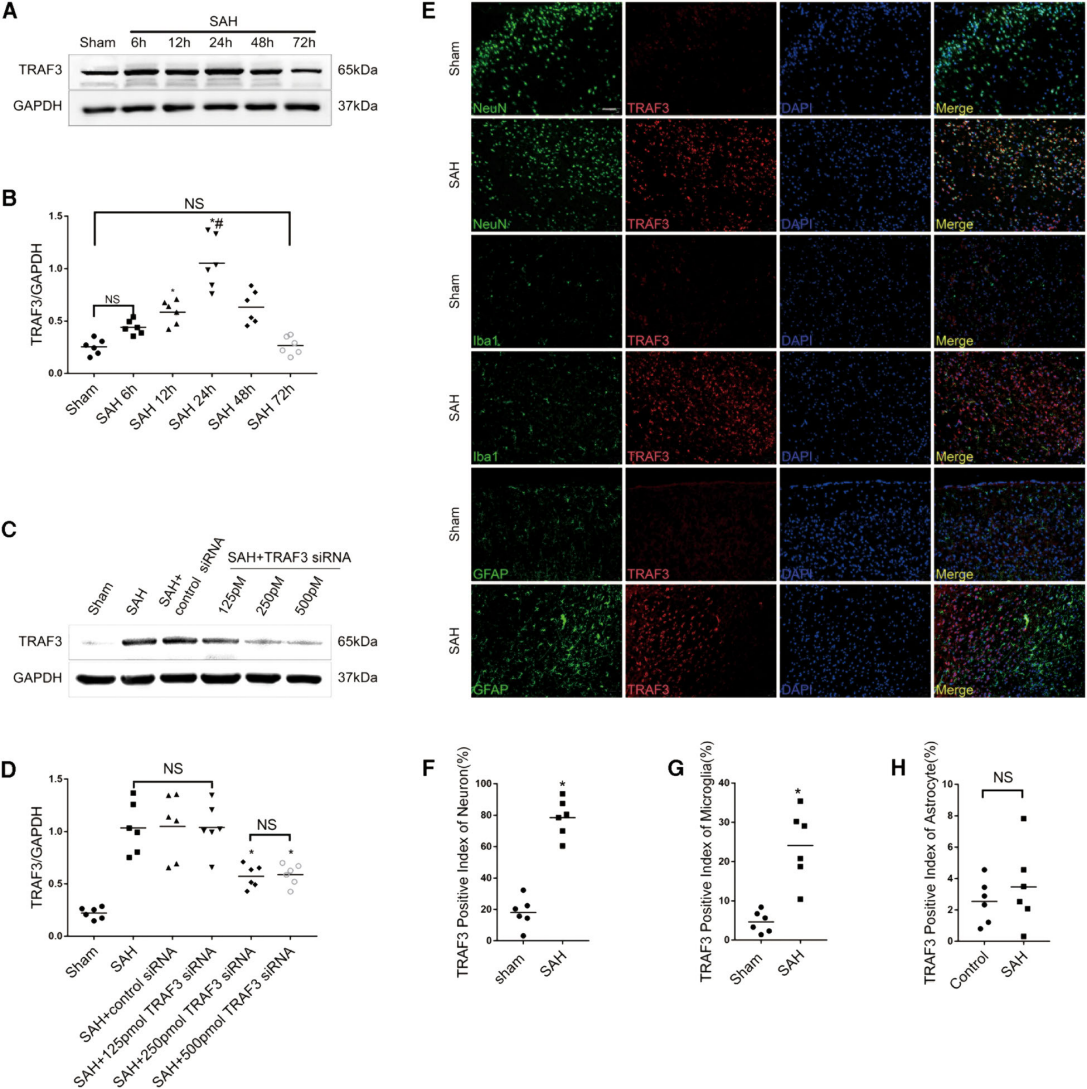
Expression and cellular distribution of TRAF3 and the effect of TRAF3 siRNA on TRAF3 expression in SAH model. **A** Western blotting analysis revealed that TRAF3 expression levels increased post-SAH. **B** Quantitative analysis of TRAF3/GAPDH. *N* = 6 mice per group. *p < 0.05 versus the sham group, ^#^P < 0.05 versus the SAH 48 h group. **C**, **D** Western blotting analysis revealed that the expression of TRAF3 in SAH + 125 pmol of TRAF3 siRNA group showed no significant change compared with SAH + control siRNA group. However, 250 and 500 pmol of TRAF3 siRNA significantly reduced the TRAF3 expression. GAPDH served as a loading control. *N* = 6 mice per group. *p < 0.05 versus the SAH group. **E** Representative double immunofluorescence images showed that TRAF3 expression was elevated 24 h following SAH and was mainly expressed in neurons, with sparse expression in microglia and no expression in astrocytes. Scale bar: 50 μm. The TRAF3 positive index of neurons (**F**), microglia (**G**), and astrocytes (**H**). *N* = 6 mice per group. *p < 0.05 versus the sham group.

We observed an upregulation of TRAF3 protein expression in temporal cortex samples of SAH mice. The western blotting analysis showed a statistically significant increase of TRAF3 protein expression at 12 h, followed by a gradual decrease after 24 h, and a return to normal levels at 72 h (Fig. 2A, B). This result was confirmed by double immunofluorescence staining. Moreover, our results showed that TRAF3 was mainly expressed in neurons, with sparse expression in microglia and no expression in astrocytes (Fig. 2E–H).

### TRAF3 siRNA reduced TRAF3 expression in SAH

To evaluate the role of TRAF3 in EBI after SAH, TRAF3 siRNA was used to reduce the expression of TRAF3 in SAH mice. The levels of TRAF3 were increased in the SAH and SAH + control siRNA groups in comparison to the sham group. Moreover, the expression of TRAF3 in the SAH + 125 pmol of the TRAF3 siRNA treatment group showed no significant difference compared to the SAH + control siRNA group. However, both 250 pmol and 500 pmol of TRAF3 siRNA did significantly downregulate TRAF3 expression in SAH. There was no significant difference between the SAH + 125 pmol of TRAF3 siRNA group and SAH + 250 pmol of TRAF3 siRNA group (Fig. 2C, D). These data led us to use 250 pmol of TRAF3 siRNA in the subsequent experiments.

### Downregulation of TRAF3 attenuated neuronal apoptosis in EBI

**Fig. 3:**
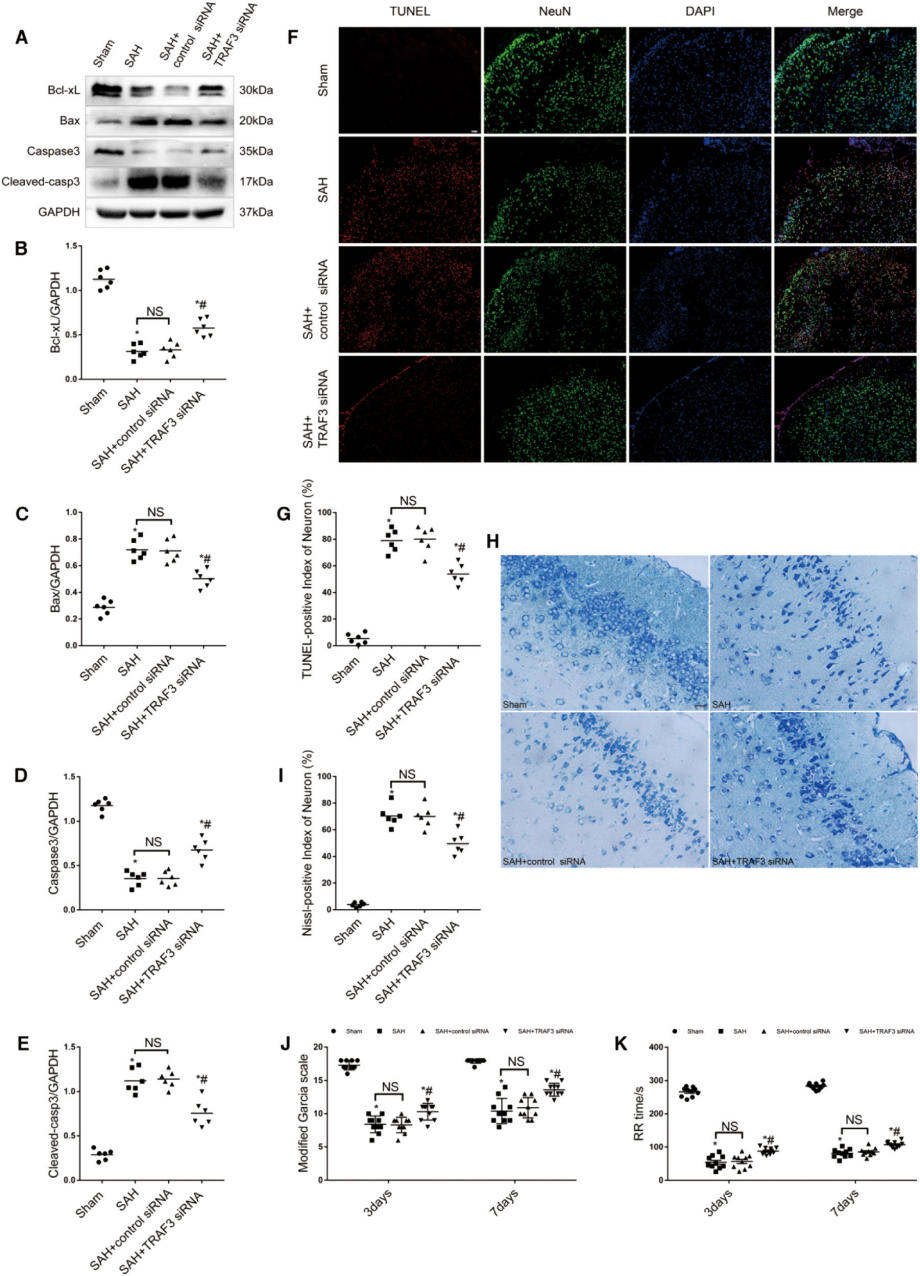
Downregulation of TRAF3 reduced neuronal apoptosis and ameliorated neurological deficits. **A** Representative western blot images of Bcl-xL, Bax, Caspase-3, and Cleaved caspase-3. Quantitative analysis showed that downregulation of TRAF3 increased the expression of Bcl-xL (**B**) and Caspase-3 (**D**), while decreased the expression of Bax (**C**) and Cleaved caspase-3 (**E**) compared with SAH and SAH + control siRNA groups. *N* = 6 per mice group. *p < 0.05 versus the sham group, ^#^*p* < 0.05 versus the SAH group. **F** Double staining of TUNEL (red) and NeuN (green); nuclei were counterstained with DAPI (blue). Scale bar: 100 μm. **G** The TUNEL-positive index of neuron in the temporal lobe was increased in the SAH and SAH + control siRNA groups, TRAF3 siRNA treatment significantly decreased the number of TUNEL-positive cells in SAH mice at 24 h post-SAH. *N* = 6 mice per group. *p < 0.05 versus the sham group, ^#^p < 0.05 versus the SAH group. **H**, **I** Nissl staining results showed that TRAF3 siRNA treatment significantly decreased the percentage of necrotic neurons at 7 days post-SAH. Scale bar: 20 μm. *N* = 6 mice per group. *p < 0.05 versus the sham group, ^#^p < 0.05 versus the SAH group. **J** Downregulation of TRAF3 could ameliorate motor coordination scores on days 3 and 7 post-SAH. **K** The RR time on days 3 and 7 post-SAH in the SAH and SAH + control siRNA groups were lower compared to the sham group. The RR time was increased by TRAF3 siRNA treatment. *N* = 10 mice per group. Data represent mean ± SEM. *p < 0.05 versus the sham group, ^#^*p* < 0.05 versus the SAH group.

Since neuronal apoptosis is a major pathological process in EBI, western blotting was used to examine protein expression of indicators of early-stage apoptosis, including Bcl-xL, Bax, caspase-3, and cleaved caspase-3 (Fig. 3A–E). The protein levels of Bcl-xL and caspase-3 were significantly decreased, and the protein levels of Bax and cleaved caspase-3 were significantly increased in the SAH and SAH + control siRNA groups compared with the sham group. However, treatment with TRAF3 siRNA significantly reduced Bax and cleaved caspase-3 expression, meanwhile, increased Bcl-xL and caspase-3 expression. Additionally, TUNEL staining was used to assess temporal neuronal apoptosis at 24 h following SAH. As shown in Fig. 3F, G, the number of TUNEL-positive cells in the temporal lobe was increased in the SAH and SAH + control siRNA groups. However, downregulation of TRAF3 decreased the number of TUNEL-positive cells in SAH mice. To further analyze whether downregulation of TRAF3 could have an effect on the longer-term improvement of neurons, Nissl staining was used to assess neuron loss at 7 d after SAH. The results showed that the percentage of necrotic neurons in SAH mice treated with TRAF3 siRNA was greatly reduced at 7 d following SAH compared to SAH and SAH + control siRNA groups (Fig. 3H, I).

### Downregulation of TRAF3 improved neurological behavior in SAH mice

The Modified Garcia score was used to evaluate the neurological function at postoperative days 3 and 7. The results showed that the Modified Garcia scores of the SAH and SAH + control siRNA groups were lower compared to the sham group. Moreover, the Modified Garcia score was significantly increased in the TRAF3 siRNA treatment group (Fig. 3J). The effects of TRAF3 downregulation on motor coordination were assessed using the rotarod test. As shown in Fig. 3K, SAH mice demonstrated significant impairment of motor coordination compared to the sham group. In contrast, downregulation of TRAF3 ameliorated motor coordination on postoperative days 3 and 7.

### Downregulation of TRAF3 inhibited SAH-induced activation of a MAPKs signaling pathway in vivo

**Fig. 4:**
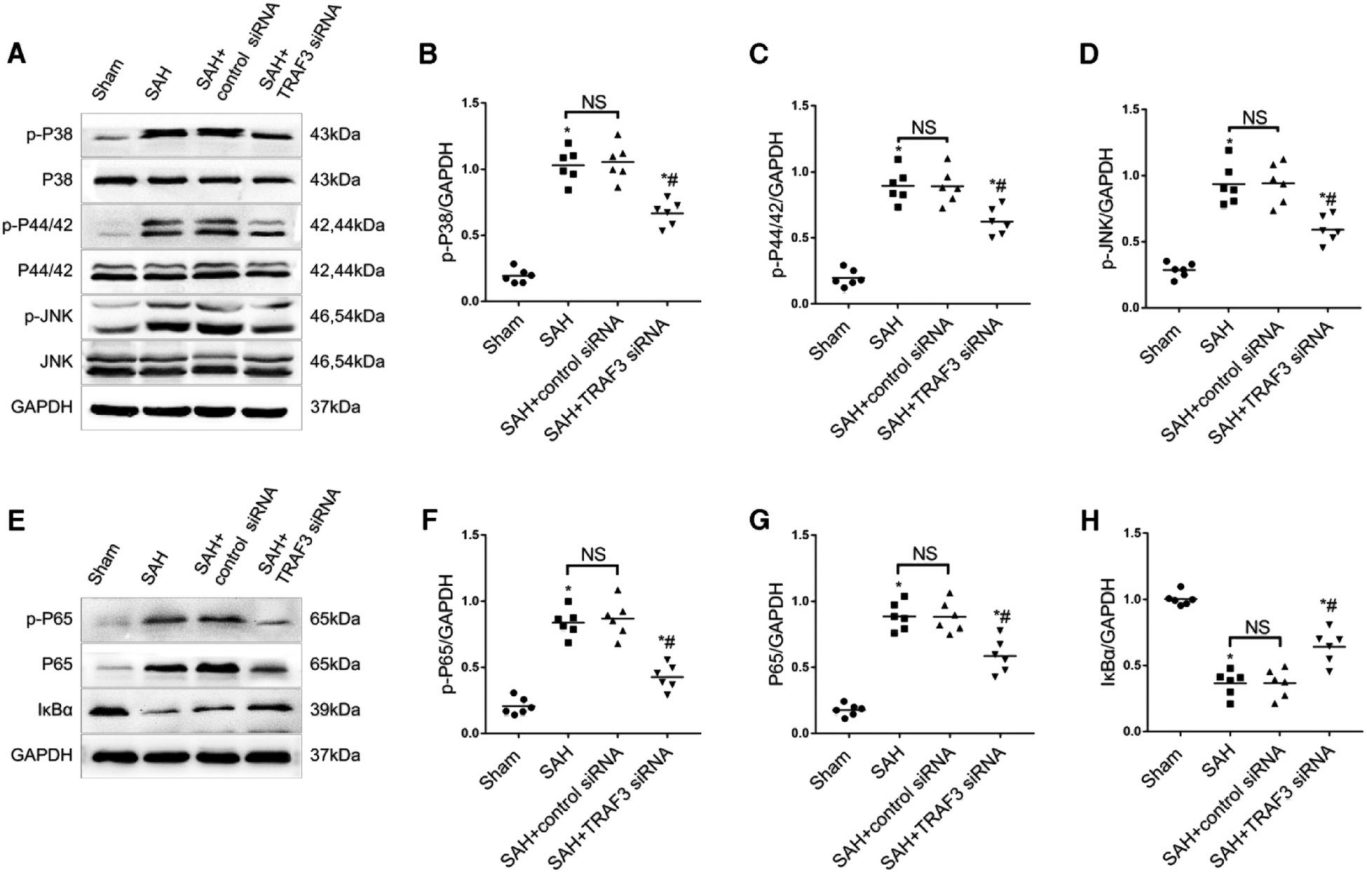
Downregulation of TRAF3 inhibited activation of MAPKs and the NF-κB signaling pathway in SAH mice. **A** Western blotting of MAPKs at 24 h after SAH. Quantitative analysis results revealed that SAH increased the expression of p-P38 (**B**), p-P44/42 (**C**), and p-JNK (**D**). TRAF3 siRNA treatment significantly reduced the expression of p-P38, p-P44/42, and p-JNK. **E** Western blotting of the NF-κB signal pathway at 24 h after SAH. The protein expression levels of NF-κB nuclear subunits p-P65 (**F**) and P65 (**G**) were increased in SAH and SAH + control siRNA groups compared with the sham group and downregulated in the SAH + TRAF3 siRNA group. The expression of IκBα (**H**) was increased in the sham group compared to the SAH and SAH + control siRNA groups; it was substantially reduced and reversed in the SAH + TRAF3 siRNA group. *N* = 6 mice per group. *p < 0.05 versus the sham group, ^#^*p* < 0.05 versus the SAH group.

To further explore the molecular mechanism of neuroprotection by downregulation of TRAF3, western blotting was used to evaluate protein levels of MAPK family members and their phosphorylation, including P38, P44/42, JNK, p-P38, p-P44/42, and p-JNK. A significant increase in the expression levels of p-P38, p-P44/42, and p-JNK was observed in the SAH group in comparison with the sham group. In addition, TRAF3 siRNA treatment significantly reduced the expressions of p-p38, p-p44/42, and p-JNK (Fig. 4A–D).

### Downregulation of TRAF3 inhibited SAH-induced activation of NF-κB signaling pathway in vivo

Previous studies have demonstrated that the NF-κB pathway has an essential role in the SAH-induced EBI pathogenesis^8,10^. To determine whether the NF-κB signaling pathway is involved in downregulation of TRAF3 following SAH, the expression of IκBα, an inhibitor of NF-κB, was investigated using western blotting. The protein level of IκBα was high in the sham group, whereas it was reduced in the SAH and SAH + control siRNA groups. However, the IκBα protein level of the SAH + TRAF3 siRNA group was higher than SAH and SAH + control siRNA groups. Moreover, the expression levels of P65 and p-P65 were increased in SAH and SAH + control siRNA groups compared with the sham group. However, P65 and p-P65 levels were also decreased in the SAH + TRAF3 siRNA group (Fig. 4E–H).

### Expression of TRAF3 in primary neuronal cells after SAH

**Fig. 5:**
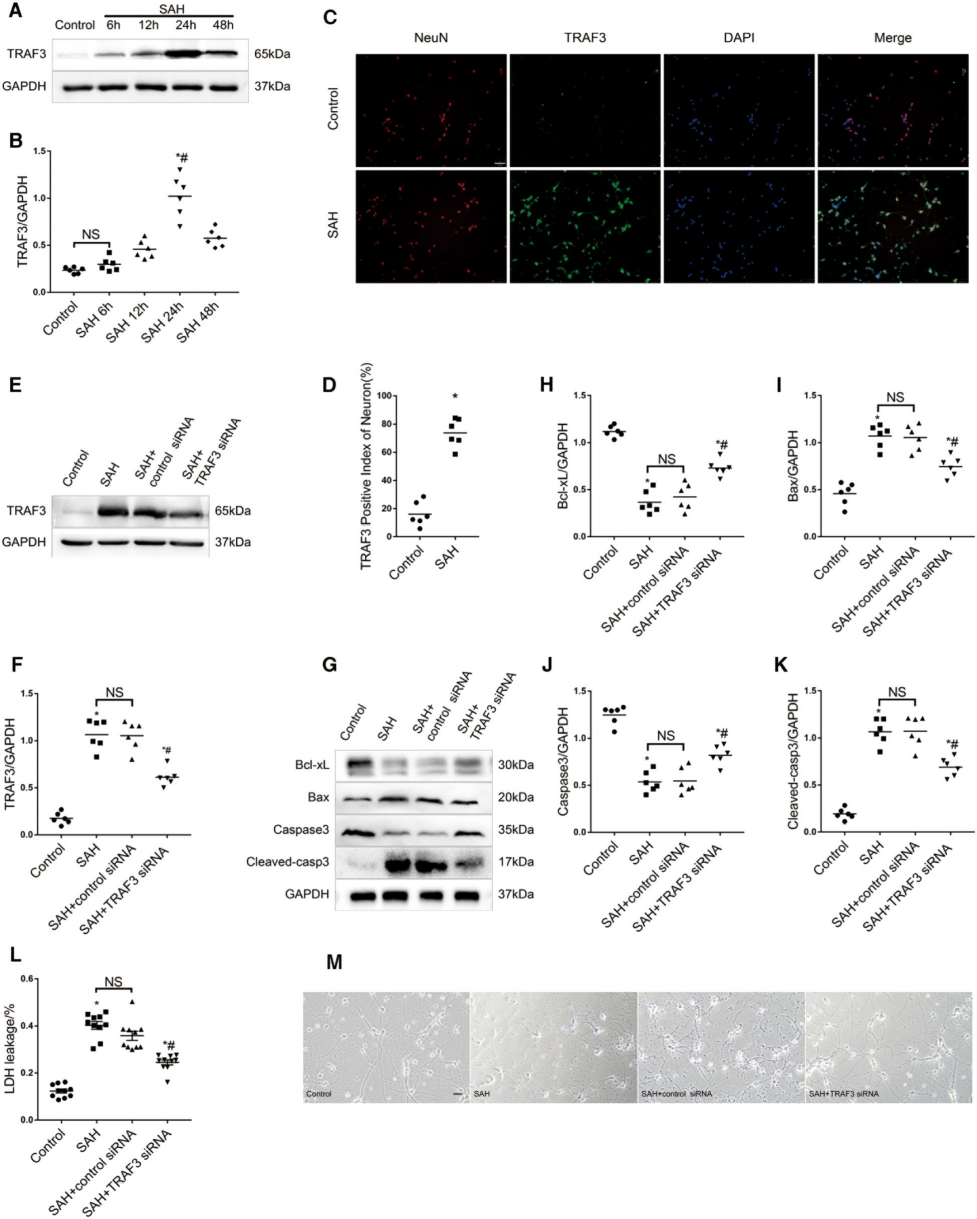
TRAF3 expression was increased and TRAF3 siRNA could alleviate neuronal apoptosis in vitro. Western blotting analysis represented that the level of TRAF3 increased significantly at 24 h following SAH (**A**, **B**). *N* = 6 wells per group. *p < 0.05 versus the control group, ^#^*p* < 0.05 versus the SAH 48 h group. **C**, **D** Overlapped images with TRAF3 and NeuN demonstrated that TRAF3 expression was elevated after SAH. Scale bar: 50 μm. *N* = 6 wells per group. *p < 0.05 versus control group. **E**, **F** Western blotting analysis showed that the level of TRAF3 could be downregulated via TRAF3 siRNA after SAH. *N* = 6 wells per group. *p < 0.05 versus the control group, ^#^*p* < 0.05 versus the SAH group. **G** Representative western blot images and quantitative analyses showed that downregulation of TRAF3 increased the expression of Bcl-xL (**H**), Caspase-3 (**J**) and decreased the expression of Bax (**I**) and C Hayakawa leaved caspase-3 (**K**) compared to SAH and SAH + control siRNA groups. *N* = 6 wells per group. *p < 0.05 versus the control group, ^#^*p* < 0.05 versus the SAH group. **L** LDH release rates demonstrated that TRAF3 siRNA treatment decreased LDH release rates compared to SAH and SAH + control siRNA groups. *N* = 10 wells per group. Data represent mean ± SEM. *p < 0.05 versus the control group, ^#^*p* < 0.05 versus the SAH group. **M** Light microscopy of neurons revealed the decrease of neuronal synapses and cell shrinkage was rescued via downregulating TRAF3. *N* = 3 wells per group. Scale bar: 20 μm.

We used hemoglobin to mimic the condition of SAH in primary neurons to determine whether TRAF3 would have an effect on SAH-induced neuronal apoptosis in vitro. Western blot analysis showed that the level of TRAF3 was upregulated at 12 h after SAH and reached a peak at 24 h following SAH (Fig. 5A, B). Additionally, this result was confirmed by double immunofluorescence staining (Fig. 5C, D).

### TRAF3 siRNA downregulated TRAF3 expression and attenuated neuronal apoptosis in SAH primary neural cells

Primary neurons were then transfected with TRAF3 siRNAs at 72 h before SAH. As shown in Fig. 5E, F, TRAF3 protein level in SAH + TRAF3 siRNA group was significantly decreased compared with the SAH group. Compared with SAH and SAH + control siRNA groups, the downregulation of TRAF3 increased the expression of Bcl-xL and caspase-3 and decreased the expression of Bax and cleaved caspase-3 (Fig. 5G–K). The lactate dehydrogenase (LDH) assay was used to assess cytotoxicity levels in primary neurons. The study showed increased LDH release rates in both SAH and SAH + control siRNA groups compared to the control group. In contrast, TRAF3 siRNA treatment decreased LDH release rates (Fig. 5L). Light microscopy of neurons showed reduced neuronal synapses and cell shrinkage in the SAH and SAH + control siRNA groups compared to the control group. When compared to SAH and SAH + control siRNA groups neuronal damage was reduced in the SAH + TRAF3 siRNA group (Fig. 5M). These results indicated that the downregulation of TRAF3 did not only improve the neuron damage in vivo SAH model but also produce similar effects in vitro.

### Downregulation of TRAF3 inhibited SAH-induced activation of MAPKs and the NF-κB signaling pathways in vitro

**Fig. 6:**
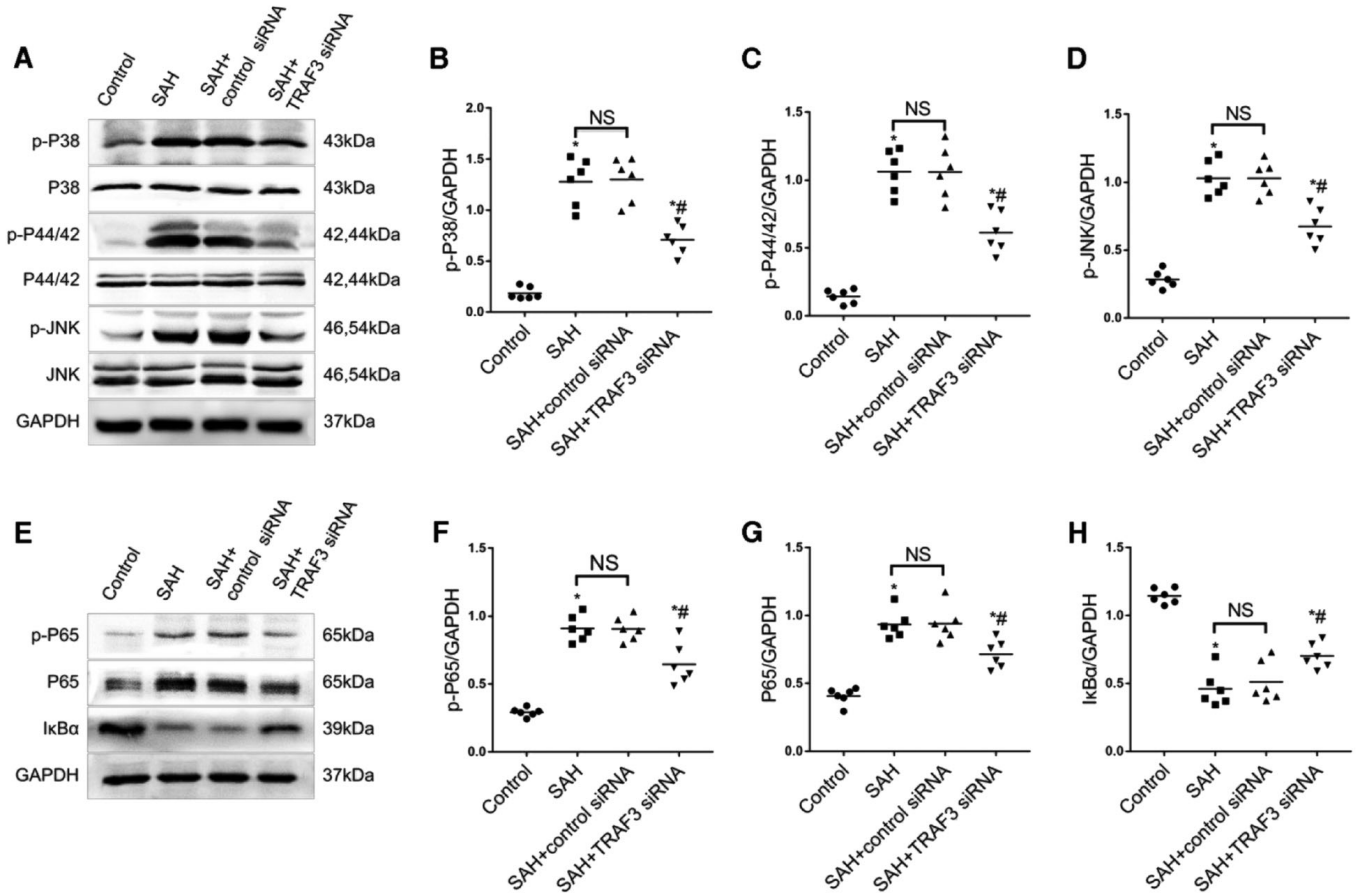
Downregulation of TRAF3 inhibited the activation of MAPKs and NF-κB signaling pathway in SAH primary neuronal cells. **A** Western blotting of MAPKs at 24 h after SAH. The P38, P44/42, and JNK expression levels were unaltered. the expression of p-P38, p-P44/42, and p-JNK increased in the SAH group compared with the control group. Quantitative analyses showed that the downregulation of TRAF3 reduced the activation of p-P38 (**B**), p-P44/42 (**C**), and p-JNK (**D**). **E** Western blotting of the NF-κB signal pathway in 24 h after SAH. The protein expression levels of NF-κB nuclear subunits p-P65 (**F**) and P65 (**G**) were increased in SAH and SAH + control siRNA groups and downregulated in the SAH + TRAF3 siRNA group. Levels IκBα was decreased after SAH, TRAF3 siRNA treatment increased the protein level of IκBα (**H**). *N* = 6 wells per group. *p < 0.05 versus the control group, ^#^*p* < 0.05 versus the SAH group.

Similarly, the expression of MAPKs and NF-κB pathway was examined using western blotting. The results showed that the downregulation of TRAF3 decreased the expression of SAH-induced p-P38, p-P44/42, and p-JNK in vitro. Besides, SAH reduced the level of IκBα and increased the level of P65 and p-P65 in vitro. Moreover, these changes in protein expression were reversed by downregulating TRAF3 (Fig. 6A–H).

### TRAF3–TAK1 interaction and phosphorylation of TAK1 is required for TRAF3-dependent neuronal apoptosis in SAH

**Fig. 7:**
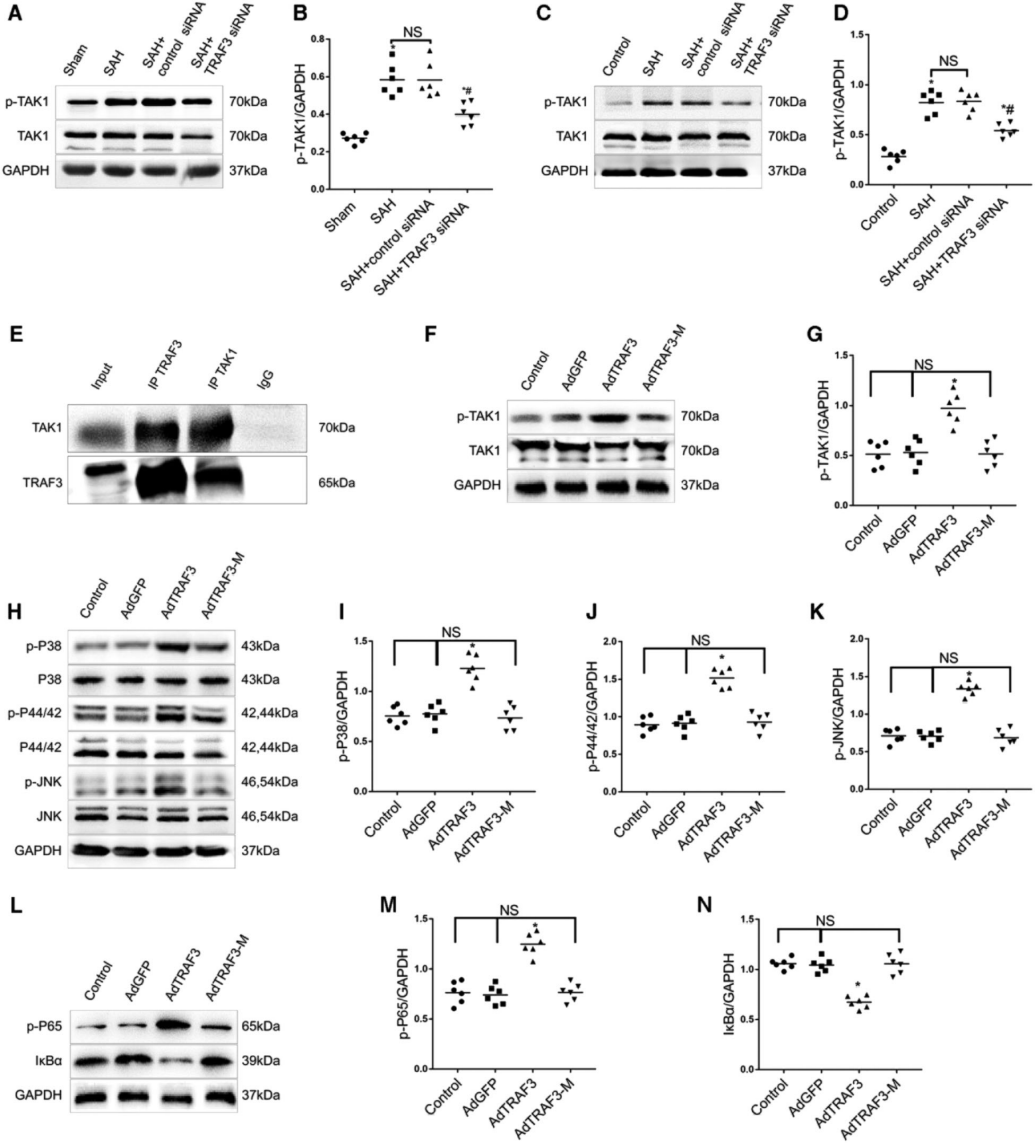
TRAF3–TAK1 interaction and phosphorylation of TAK1 are required for TRAF3-dependent neuronal apoptosis in SAH. Western blot analysis revealed that TRAF3 siRNA reduced the phosphorylation of TAK1 after SAH both in vivo (**A**, **B**) and in vitro (**C**, **D**). *N* = 6 mice or wells per group. *p < 0.05 versus the control group, ^#^*p* < 0.05 versus the SAH group. **E** Co-immunoprecipitation showed that the expression of TRAF3 could interact with TAK1 in SAH primary neural cells. **F**, **G** Western blotting analysis showed that AdTRAF3-M failed to increase the expression of p-TAK1 in primary neural cells while AdTRAF3 succeeded. **H**–**N** Western blotting analysis showed that the activation of MAPKs and NF-κB signaling was potentiated by AdTRAF3, but abrogated by AdTRAF3-M. *N* = 6 wells per group. *p < 0.05 versus the control group.

TRAF3 has been reported as a critical upstream regulator of MAPKs and NF-κB pathways via regulation of TAK1^14^. Therefore, we assumed that TRAF3 facilitated SAH-induced neuronal death via direct phosphorylation of TAK1. As respected, the western blotting results revealed that downregulation of TRAF3 significantly inhibited the phosphorylation of TAK1 after SAH, both in vivo and in vitro (Fig. 7A–D).

We next investigated whether a direct interaction existed between TRAF3 and TAK1. Co-immunoprecipitation was observed between TRAF3 and TAK1 in SAH primary neural cells (Fig. 7E). Since the 267–376 amino acid (aa) domain of TRAF3 is responsible for the capacity to interact with TAK1^14,26,27^, we further constructed AdTRAF3-M to transfect primary neural cells. Compared with the control adenoviral vectors infection group, the AdTRAF3 infection significantly increased the p-TAK1 expression, whereas the AdTRAF3-M infection did not enhance the p-TAK1 expression (Fig. 7F, G). Furthermore, the primary neuronal cells were infected with AdTRAF3 and AdTRAF3-M. As expected, the activation of MAPKs and NF-κB signaling was potentiated by AdTRAF3, but abrogated by AdTRAF3-M (Fig. 7H–N). Collectively, these results suggest that TRAF3 regulates neuronal apoptosis in SAH probably depends on the TRAF3–TAK1 interaction and activation of TAK1.

### TRAF3 expression in the cortex increased in brain tissue of patients with SAH

**Fig. 8:**
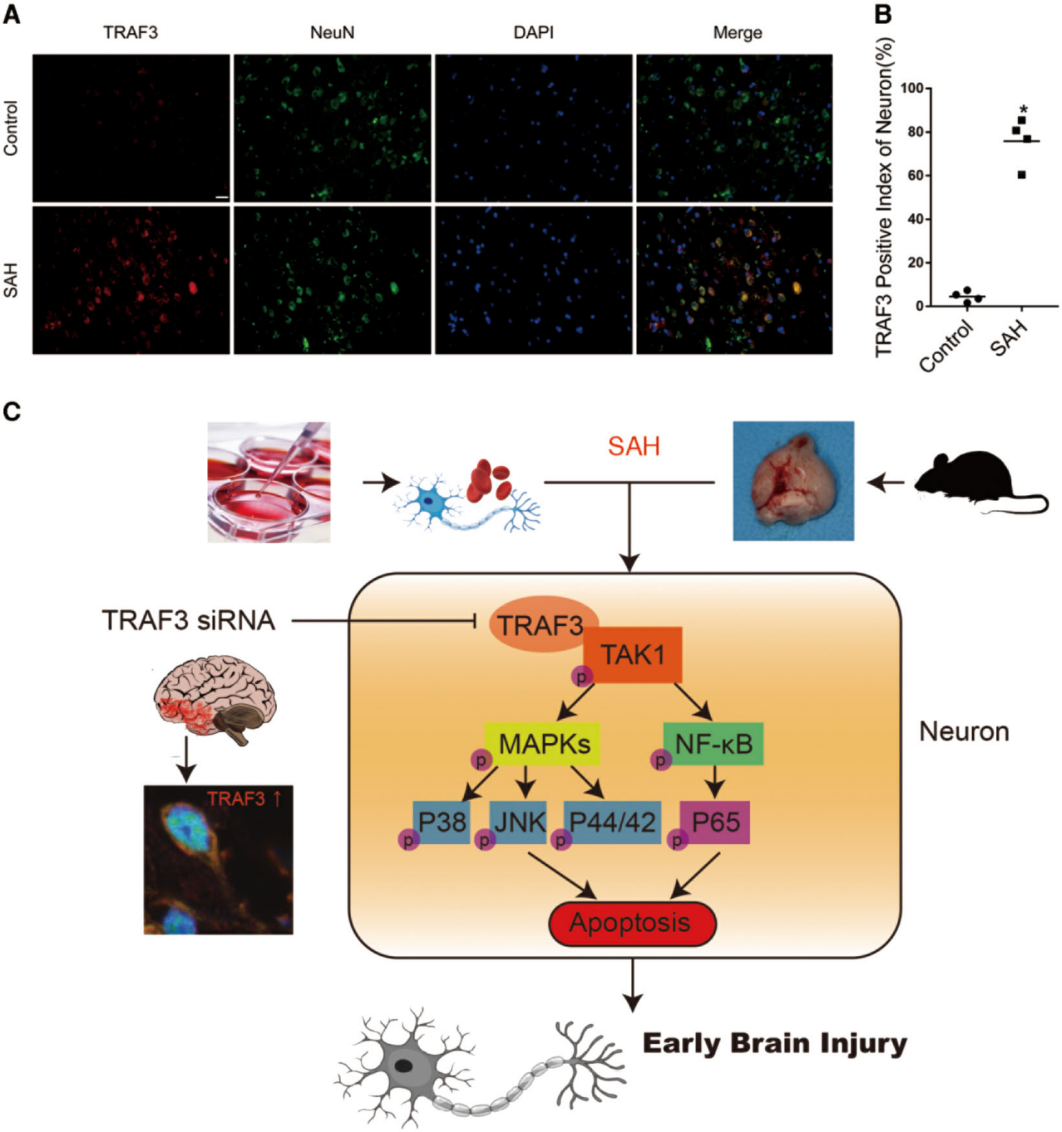
TRAF3 expression in the cortex increased in brain tissue of patients with SAH and the possible mechanisms of the antiapoptotic role of TRAF3 in SAH-induced EBI. **A** Immunofluorescence staining results confirmed that TRAF3 (red) expression was elevated after SAH and was mainly expressed in neurons (green). Scale bar: 50 μm. **B** The TRAF3 positive index of neurons. *N* = 4 people per group. *p < 0.05 versus control group. **C** Schematic illustrating the possible mechanisms of antiapoptotic role of TRAF3 in EBI after SAH.

To initially validate the feasibility of TRAF3 as an early therapeutic target for EBI in patients with clinical SAH, we evaluated TRAF3 expression levels in brain tissue excised from patients who had experienced SAH using double immunofluorescence staining. Accordingly, our results demonstrated that TRAF3 expression levels were elevated following SAH and it was mainly expressed in neurons (Fig. 8A, B).

## Discussion

As has been shown in a large number of studies, complex molecular mechanisms and multiple pathophysiological events are involved in EBI induced by SAH^6^. Consequently, using a single target that could regulate multiple signaling pathways in SAH-induced EBI may serve as a promising strategy. In the present study, we have demonstrated the following (Fig. 8C): (1) the expression of TRAF3 was upregulated after SAH both in mouse cortex and in primary cortical neurons; (2) after silencing TRAF3 gene by siRNA, the activation of TAK1/MAPK and NF-κB signaling pathway were inhibited; (3) the downregulation of TRAF3 was sufficient to reduce neuronal apoptosis both in vivo and in vitro following SAH; (4) the downregulation of TRAF3 alleviated the neurological deficits of SAH mice as demonstrated by the reduction in the Modified Garcia scores; (5) the TRAF3 expression was upregulated in neurons in patients who had experienced SAH. Considering the above findings, TRAF3 may serve as an ideal therapeutic target in SAH-induced EBI.

TRAF3 participates in modulating diverse cellular functions via its zinc finger motifs in the N-terminal interacting with adaptors, receptors, kinases, and regulatory proteins^28–30^. It has been reported that neuronal TRAF3 deficiency inhibited neuronal apoptosis after brain ischemic stroke and spinal cord injury^14,31,32^. Moreover, neuronal apoptosis involving multiple pathophysiological events in SAH-induced EBI is the main predictor of poor prognosis in SAH^4,5^. In the current study, we report that the expression of TRAF3 was significantly increased following SAH. In addition, TRAF3 was mainly expressed in neurons, with sparse expression in microglia and no expression in astrocytes. Importantly, TRAF3 siRNA treatment inhibits neuronal apoptosis by downregulating Bax and cleaved caspase-3 expression, while upregulating Bcl-xL and caspase-3 expression. Furthermore, we demonstrated that downregulating TRAF3 could reduce neuronal loss and ameliorate neurological deficits after SAH. Therefore, TRAF3 has a vital role in EBI. In the current study, we further observed that TRAF3 was also upregulated in microglia after SAH. When activated, microglia will secrete proinflammatory cytokines and reactive oxygen species, which disrupt the blood–brain barrier and induce secondary neuronal injury after SAH^33^. Thus, it remains to be studied whether TRAF3 has a functional role in EBI through modulating microglial activation.

TRAF3 has been demonstrated to promote neuronal apoptosis via the NF-κB pathway^34,35^. NF-κB regulates cellular pathophysiological processes, such as the activity of apoptotic mediators and inflammatory cytokines in SAH-induced EBI^15^, therefore, it has been considered to be a potential therapeutic target in EBI prevention^36^. In the current study, our research demonstrates that the downregulation of TRAF3 inhibits the activation of IκBα–P65 axis. Based on these observations, we concluded that the neuroprotective effect of TRAF3 could be partially mediated through the regulation of the NF-κB pathway.

The MAPKs, including ERK, p38 MAPK, and JNK, have a central role in stress-induced cell death as well as in apoptosis. TRAF3 also regulates apoptosis using MAPK signaling pathways^14,37,38^. In ischemic stroke, TRAF3 overexpression resulted in JNK phosphorylation, leading to neuronal apoptosis^14^. In hepatic ischemic injury, the expression of p-JNK was elevated by TRAF3 upregulation and suppressed by TRAF3 deletion^26^. Further, JNK and P38 have been confirmed to have an important role in the development of EBI after SAH^11,15^. In the current study, we found that the downregulation of TRAF3 led to decreased JNK and P38 phosphorylation, which further regulated downstream effectors.

Our previous study demonstrated that TAK1, a member of the MAP3Ks family, acts as a key regulator of NF-κB and the MAP kinases JNK and P38 and modulates post-SAH apoptosis^15^. Targeting TAK1 may have great potential to alleviate the EBI following SAH. Despite the important role of TAK1 in EBI, the mechanism by which TAK1 kinase is activated remains poorly understood. Previous studies showed that TRAF3 could negatively regulate TAK1/MAPK immune signaling^39^. In contrast, TRAF3 can stimulate TAK1 phosphorylation in ischemic stroke and hepatic ischemic injury^14,26,27^. The differences in these findings could be attributed to variations in disease models, animals, cell types, and stimuli involved. In the current study, we demonstrated that the downregulation of TRAF3 decreased TAK1 phosphorylation in SAH-induced EBI, suggesting that it has a pivotal role in the regulation of TAK1 activity.

Further, we investigated how TRAF3 regulated the phosphorylation of TAK1 in SAH. The co-immunoprecipitation result indicates that TRAF3 directly interacts with TAK1 in SAH. Previous studies have shown that the interaction between the 267–376 amino acid (aa) domain of TRAF3 and the 481–579 aa domain of TAK1 contributes to the phosphorylation and activation of TAK1^14,27^. In our study, AdTRAF3-M (adenoviruses carrying sequences encoding mouse TRAF3 with a mutant 267–376 aa domain) failed to increase the expression of p-TAK1 in primary neural cells while AdTRAF3 succeeded. Besides, AdTRAF3 but not AdTRAF3-M could potentiate the activation of MAPKs and NF-κB signaling pathways. Therefore, we deduce that the 267–376 amino acid (aa) domain of TRAF3 is essential for its capacity to interact with TAK1 in SAH.

Despite numerous basic discoveries in SAH animal research, few have turned out to be effective in clinical trials on humans. Moreover, oftentimes, animal research findings do not translate into human medicine due to incomplete replication of human pathophysiology^40–42^. Here, we have demonstrated that TRAF3 expression was elevated after clinical SAH and was mainly expressed in neurons, suggesting that downregulation of TRAF3 may have potential clinical application in the treatment of EBI. Considering that only patients with very severe SAH requiring surgery could provide brain tissue in our research, further studies are warranted to determine whether TRAF3 expression is increased in less severe SAH patients.

In conclusion, our research has revealed that downregulation of TRAF3 inhibited the activation of MAPKs and NF-κB pathways via inhibiting the phosphorylation of TAK1, which effectively reduced neuronal apoptosis and neurological deficits in SAH. Thus, TRAF3 is a novel mediator of neuronal apoptosis in EBI following SAH. More importantly, we demonstrated that TRAF3 expression was elevated in human neurons following SAH. Therefore, TRAF3 may serve as a potential therapeutic target for improving prognosis in SAH patients.
